# Impact of C3 Vertebra-Based Sarcopenia and Clinical Factors on Postoperative Complications in Oral Cancer Patients

**DOI:** 10.3390/cancers18061004

**Published:** 2026-03-20

**Authors:** Comert Sen, Mehmet Furkan Kurşun, Onur Ozçelik, Sinan Seyrek, Murat Ulusan, Bora Başaran, Ismet Aslan

**Affiliations:** 1 Department of Otorhinolaryngology-Head and Neck Surgery, Istanbul Faculty of Medicine, Istanbul University, Istanbul 34091, Turkey; 2 Department of Radiology, Istanbul Faculty of Medicine, Istanbul University, Istanbul 34091, Turkey

**Keywords:** oral squamous cell carcinoma, sarcopenia, postoperative complications, head and neck neoplasms, body composition, skeletal muscle index, risk stratification

## Abstract

**Background/Objectives**: Recent meta-analyses have established that factors such as sarcopenia, male sex, and low serum albumin significantly correlate with increased postoperative complications in head and neck surgery, with routine neck computed tomography (CT) at the third cervical vertebra (C3) serving as a practical tool for muscle mass assessment. This study aimed to confirm the prognostic value of C3-based sarcopenia and specific clinical comorbidities in predicting early postoperative complications in patients with oral squamous cell carcinoma (OSCC). **Methods**: A retrospective cohort study was conducted on 167 patients undergoing primary surgery for OSCC. Sarcopenia was assessed using the C3-vertebra skeletal muscle index (SMI) derived from routine preoperative neck CT scans. Clinical and surgical variables, including preoperative serum albumin levels, comorbidities, and flap reconstruction types, were evaluated. A priori multivariate logistic regression models were utilized to identify independent predictors of surgical site and pulmonary and total complications (Clavien–Dindo classification) within 30 days. **Results**: The overall complication rate was 51%. Multivariate analysis revealed that sarcopenia (aOR: 3.26; 95% CI: 1.11–9.56), male sex (aOR: 3.48; 95% CI: 1.11–10.85), coronary artery disease (CAD) (aOR: 4.30; 95% CI: 1.21–15.36), and free-flap reconstruction (aOR: 15.06; 95% CI: 2.47–92.01) were robust independent predictors of total complications. Male sex (aOR: 4.17; 95% CI: 1.51–11.58) and preoperative hypoalbuminemia (<3.5 g/dL) (aOR: 3.43; 95% CI: 1.20–9.82) were independent predictors of surgical site complications, while regional flap reconstruction was independently associated with pulmonary complications (aOR: 5.97; 95% CI: 1.38–25.97). **Conclusions**: Sarcopenia, male sex, CAD, and flap reconstruction type are strong independent predictors of postoperative morbidity in OSCC. These findings advocate for “opportunistic screening” of muscle mass via routine preoperative neck CT, alongside rigorous cardiovascular profiling, to identify high-risk phenotypes for targeted perioperative optimization.

## Simple Summary

Surgery for oral cancer carries a high risk of complications. Large-scale studies have shown that factors such as male sex, low serum albumin, and physical frailty—specifically low muscle mass (sarcopenia)—correlate with increased postoperative risks in head and neck cancer. This study aimed to confirm these findings specifically in oral cavity cancer by analyzing muscle mass measured from routine, preoperative neck computed tomography (CT) scans at the third cervical vertebra (C3) level. Our retrospective analysis of 167 patients supports the existing literature, confirming that sarcopenia, male sex, and coronary artery disease are strong predictors of post-surgical problems. Clinicians can integrate muscle mass assessment into the preoperative evaluation using existing neck CT scans without additional cost or radiation.

## 1. Introduction

Patients with oral cancer frequently have impaired oral intake, leading to malnutrition and accelerated muscle loss [1,2,3]. Preoperative risk stratification helps assess this, impacting postoperative morbidity [4,5]. Physical frailty, specifically sarcopenia (defined as severe depletion of skeletal muscle mass), has emerged as a critical prognostic factor in head and neck cancer [6,7,8].

Recent large-scale systematic reviews and meta-analyses have robustly established that sarcopenia is an independent predictor of adverse outcomes in surgically treated head and neck squamous cell carcinomas [9]. The extensive literature, including meta-analyses by Xie et al. [10], Luo et al. [11], Huang et al. [12], and Yang et al. [13], consistently demonstrates that preoperative sarcopenia significantly increases the risk of 30-day mortality, pneumonia, surgical site infections, and overall postoperative complications, while simultaneously reducing long-term survival [14]. Furthermore, it has been shown that morphological frailty markers, such as sarcopenia, can be stronger predictors of wound complications than traditional markers like preoperative serum albumin levels or body mass index (BMI) [15,16].

Sarcopenia can be quantified opportunistically through routine preoperative cross-sectional imaging without requiring additional scans [17]. While the third lumbar (L3) vertebra is the conventional landmark, numerous recent studies have validated the use of routine neck computed tomography (CT) to assess skeletal muscle cross-sectional area at the level of the third cervical (C3) vertebra, providing a highly practical tool for head and neck surgeons [15,18,19,20].

This retrospective cohort study aims to support and build upon the existing literature by confirming the prognostic value of C3-based sarcopenia, alongside relevant clinical comorbidities, in predicting early postoperative complications specifically within a cohort of patients with oral squamous cell carcinoma (OSCC) [2,21].

## 2. Materials and Methods

This retrospective single-center study included 167 patients who underwent primary surgical resection for OSCC between January 2020 and June 2025. Patients with a history of head and neck radiotherapy, chemotherapy, recurrent tumors, or those under 18 years of age were excluded. The study protocol was approved by the Institutional Clinical Research Ethics Committee (Approval No: 2024/1801).

Medical records were reviewed to identify postoperative complications occurring within 30 days of surgery. Complications were graded according to the Clavien–Dindo classification system (minor: grades 1–2; major: grades 3–5) [22]. The dependent variables were defined as surgical site complications (e.g., wound infection, dehiscence, fistula, and flap necrosis), pulmonary complications (e.g., pneumonia, atelectasis, pleural effusion), or the combination of both, referred to as total complications. Due to inconsistent documentation of minor non-pulmonary medical events in outpatient records, systemic complication analysis was strictly limited to rigorously documented pulmonary complications.

Clinical and demographic data, including age, sex, body mass index (BMI), tumor characteristics (pT, pN, primary site), and surgical details (tracheotomy, neck dissection, flap reconstruction type), were collected. Continuous surgical variables such as exact operative duration and precise intraoperative blood loss were not systematically available in the retrospective database and were therefore excluded from the analysis. Based on their established prognostic relevance in major surgery, specific critical comorbidities—namely coronary artery disease (CAD) and chronic obstructive pulmonary disease (COPD)—were also recorded for risk stratification

To assess sarcopenia, the cross-sectional areas of the paravertebral and sternocleidomastoid muscles were measured on preoperative neck CT scans at the level of C3. All CT images were obtained within 45 days prior to surgery using a standardized Hounsfield Unit (HU) threshold range of −29 to +150. The total skeletal muscle area at the third lumbar vertebra (L3) level was estimated using the previously validated Swartz formula [18]. The skeletal muscle index (SMI) was then calculated by normalizing this estimated L3 muscle area for patient height (cm^2^/m^2^). Following the established literature for head and neck squamous cell carcinoma cohorts, a singular cutoff value of SMI < 43.2 cm^2^/m^2^ was utilized to define the presence of sarcopenia [19,20,23,24,25].

Statistical analyses were performed using IBM SPSS Statistics, Version 28.0 (IBM Corp., Armonk, NY, USA). Descriptive statistics summarized patient characteristics. To address the law of multiplicity and avoid over-testing individual variables, we deliberately bypassed extensive univariate screening. Instead, a multivariate logistic regression model was constructed directly. Variables included in the final multivariate model (such as sarcopenia, sex, CAD, and preoperative albumin) were selected a priori based on strong clinical rationale and the established literature regarding postoperative morbidity in head and neck surgery [4,10,11,12,13,26,27]. Multicollinearity among the included variables was verified using the Variance Inflation Factor (VIF), with all values remaining well within acceptable limits (<2.5). A *p*-value of <0.05 was considered statistically significant.

## 3. Results

### 3.1. General Information

One hundred and sixty-seven patients (99 male, 68 female) were included in the study. Tumor localization was heterogeneous, with approximately half of the cases involving the oral tongue. Flap reconstruction was performed in 114 patients (68%), of whom 87 received regional flaps and 27 received free flaps. The overall prevalence of sarcopenia in the study cohort was 50% (*n* = 84). Sarcopenia was significantly associated with female sex, age ≥ 70 years, BMI < 20 kg/m^2^, lower serum albumin levels (<3.5 g/dL), and chronic obstructive pulmonary disease (COPD) (*p* < 0.05 for all) (Table 1).

Surgical site complications were observed in 43% of patients, and pulmonary complications in 23%. Overall, 51% experienced at least one complication, and 32% experienced major complications (Clavien–Dindo grade ≥ 3). Surgical site complications included 33 seroma, 25 hematoma, 26 wound infection, flap necrosis (17 marginal, 11 partial, 6 total), 46 wound dehiscence, and 10 fistulae. Pulmonary complications included 23 pneumonia, 20 atelectasis, 15 pleural effusion, 5 pulmonary embolism and 4 pneumothorax. The average hospital stay was 13 days. Two patients died within 30 days of surgery, and 10 patients required readmission within one month.

### 3.2. Statistical Analysis

We evaluated risk factors using multivariate logistic regression (avoiding issues of the law of multiplicity in a univariate analysis) with variables selected based on the literature. Multivariate analysis showed distinct independent risk factors for different complication types. Surgical complications were significantly associated with male sex, intraoperative tracheotomy, and preoperative hypoalbuminemia (<3.5 g/dL) (*p* < 0.05). Regional flap reconstruction was the only significant independent predictor of pulmonary complications (*p* < 0.05). Male sex, CAD, flap reconstruction (regional or free flap) and sarcopenia were all robustly and independently associated with development of complications (*p* < 0.05) (Table 2).

## 4. Discussion

This retrospective cohort study evaluated the risk factors affecting surgery complications, pulmonary complications, and both (total complications) occurring within 30 days after surgery in patients with OSCC. A multivariate analysis shows that sarcopenia defined by a C3-based skeletal muscle index is a robust independent predictor of total complications (OR: 3.26). This finding supports the prior literature, including recent systematic reviews and meta-analyses by Xie [10], Luo [11], and Huang [12], which establishes that sarcopenia strongly correlates with higher complication rates and reduced survival in oral cavity cancer [13,14].

Our data confirm that measuring skeletal muscle depletion at the C3 level using routine preoperative neck CT scans, which is already done for tumor evaluation, is a practical effective method to assess risk [15,18,20]. Although our radiological approach (C3-based CT) relies on predictive formulas compared to direct L3 measurements, the consistency of our findings with the broader literature underscores that C3-derived sarcopenia remains a valuable tool [16]. Beyond muscle mass, emerging evidence highlights the prognostic importance of muscle quality, such as myosteatosis and the albumin–myosteatosis gauge, which have been shown to impact cancer prognosis--another perioperative assessment parameter [28,29].

Our analysis identified male sex and coronary artery disease (CAD) as significant independent risk factors for postoperative complications. This is consistent with larger analyses in the literature, which have repeatedly demonstrated that male sex and CAD are intrinsic risk factors for adverse postoperative outcomes in major head and neck surgery [4,26,30,31,32]. Furthermore, the type of reconstruction significantly influenced outcome profiles. Free-flap reconstruction was unsurprisingly associated with a higher risk of total complications, while regional flaps were significantly associated with pulmonary morbidity, consistent with the literature [33,34,35].

## 5. Limitations

This study has several limitations inherent to a retrospective single-center design. First, we deliberately utilized a singular literature-based cutoff for sarcopenia (43.2 cm^2^/m^2^) rather than sex-specific thresholds. While this maintains strict methodological consistency with previous key HNSCC cohorts (e.g., Orzell et al. and Ansari et al.) to ensure comparability [19,20,23,24,25], we acknowledge it deviates from general geriatric definitions (e.g., EWGSOP2) [36]. This uniform cutoff likely introduces classification bias by overestimating sarcopenia in female patients. Future prospective studies should evaluate sex-adjusted cutoffs in this population.

Our evaluation of systemic complications was limited to pulmonary events, as incomplete outpatient records for other minor medical issues prevented a comprehensive analysis. This exclusion likely underestimates the true total morbidity rate and introduces a degree of outcome misclassification bias.

While flap reconstruction emerged unsurprisingly as an independent predictor of complications, continuous variables such as operative time, defect size, and blood loss were not systematically available in our database. Hence we could not control for these surgical complexity factors, which likely act as confounders in our multivariate model. Finally, our assessment of muscle status was purely morphological; the lack of functional data, such as handgrip strength or gait speed, precludes a diagnosis of “severe sarcopenia” according to the latest consensus guidelines [10].

## 6. Conclusions

This study supports the literature that demonstrates that sarcopenia assessed via the C3-based skeletal muscle index correlates with postoperative complications in patients with OSCC. Integrating neck CT-based sarcopenia assessments in patients who have had a CT into the preoperative workflow adds no expense and supplements other variables (low albumin, recent weight loss) that would be expected to be correlated with sarcopenia.

## Figures and Tables

**Table 1:** Basic characteristics by sarcopenia status.

Patient Population	Sarcopenia (n = 84)	Non-Sarcopenia (n = 83)
Age ≥ 70 *	48	22
Male, Female *	28; 56	12; 71
Chronic obstructive pulmonary disease (COPD) *	8	1
Coronary artery disease (CAD)	16	12
Smoking	37	49
Alcohol	14	20
BMI: <20, 20–25, >25 *	13; 11; 38	3; 24; 58
Hb < 12 g/dL	18	11
Albumin < 3.5 g/dL *	21	7
Tumor location: Tongue	43	42
Floor of mouth	5	5
Buccal mucosa	10	4
Retromolar trigon	12	9
Hard palate	4	5
Alveolar ridge	6	12
Lips	4	6
Primary stage: T1, T2, T3, T4	10; 15; 24; 32	15; 22; 16; 30
Nodal stage: N0, N1, N2, N3	40; 7; 16; 21	43; 10; 15; 15
Neck dissection: no, unilateral, bilateral	6; 20; 42	2; 34; 33
Tracheotomy	58	53
Flap: none, regional, free flap	22; 49; 13	31; 38; 14

* Statistically significant (*p* < 0.05). Abbreviations: n: number, BMI: body mass index; Hb: hemoglobin.

**Table 2:** Multivariate logistic regression for postoperative complications.

Variables	Surgical Site Complications	Pulmonary Complications	Total Complications
	aOR (95% CI)	aOR (95% CI)	aOR (95% CI)
Male Sex	4.17 (1.51–11.58) *	-	3.48 (1.11–10.85) *
CAD (Yes)	1.73 (0.64–4.68)	2.93 (0.81–10.65)	4.30 (1.21–15.36) *
Tracheotomy (Yes)	3.63 (1.33–9.87) *	-	1.15 (0.26–5.05)
Free flap	-	4.19 (0.77–22.75)	15.06 (2.47–92.01) *
Regional flap	-	5.97 (1.38–25.97) *	6.97 (1.53–31.80) *
Albumin < 3.5 g/dL	3.43 (1.20–9.82) *	-	2.32 (0.67–6.77)
Sarcopenia (Yes)	2.12 (0.81–5.55)	2.03 (0.76–5.43)	3.26 (1.11–9.56) *

* Statistically significant independent predictor (*p* < 0.05). Abbreviations: aOR: adjusted odds ratio; CI: confidence interval; CAD: coronary artery disease. Note: Empty cells (-) indicate variables that were not forced into the final adjusted model for that specific complication subset to avoid overfitting.

## Data Availability

The data presented in this study are available on request from the corresponding author. The data are not publicly available due to privacy and ethical restrictions regarding patient confidentiality.
